# Risk prediction for individual patients and the pitfalls of selecting an optimal prediction model: do not judge a model by its c-statistic

**DOI:** 10.1007/s10654-026-01378-2

**Published:** 2026-05-27

**Authors:** Jan Willem van Dalen

**Affiliations:** 1 Department of Neurology, Donders Institute for Brain, Cognition, and Behaviour, Radboud University Medical Center, Geert Grooteplein Zuid 10, Nijmegen, 6525 GA the Netherlands; 2 Department of Public & Occupational Health, Amsterdam UMC, University of Amsterdam, Meibergdreef 9, Amsterdam, 1105 AZ the Netherlands

## Abstract

Researchers often report the area under the curve (AUC/c-statistic), Brier-index (BI), and explained variation (R^2^) to assess risk prediction model performance. This may be inappropriate for scores to estimate individual patient risks because population characteristics like risk score distributions may influence these performance metrics. This study assessed performance values for a fully accurate risk score under different distributions. A risk score ranging 0-100% was simulated for 100,000 individuals with 1000 bootstraps under six distributions: normal, uniform, bimodal, extreme bimodal, uniform ascending and uniform descending. Outcomes were simulated with full accuracy: 1% of individuals with a score of 0.01 had an outcome, 2% with 0.02 etc. Values for the AUC, BI, BI scaled (BS), and R^2^ were calculated, and calibration plots made. Sensitivity analyses included narrowing risk score range and reducing score granularity. For a normally distributed score, performance indices did not exceed values generally considered poor (AUC = 0.67, BI = 0.23, BS = 0.08, R^2^ = 8%). Values were best for an extreme bimodal distribution (AUC = 0.93, BI = 0.10, BS = 0.61, R^2^ = 61%). Performance indices for the other distributions were moderate (AUC = 0.80–0.83, BI = 0.17–0.18, BS = 0.25–0.33, R^2^ = 25–33%). Calibration plots were perfect for all distributions. Values worsened with narrowing score ranges. Reducing granularity had marginal effects. Risk prediction model performance values depend strongly on risk distributions. They may not exceed values generally considered poor/moderate in many common risk distributions. When assessing patient risk prediction models, investigators and clinicians interested in the accuracy of risk estimations may prioritize calibration and net benefits over commonly used metrics like the AUC and BI.

**Supplementary Information:**

The online version contains supplementary material available at 10.1007/s10654-026-01378-2.

## Introduction

In general medicine, there is great interest in prediction models for risk estimation. These models use personal characteristics to calculate an individual’s risk of experiencing a serious clinical event (e.g. myocardial infarction, stroke or death) within a certain timeframe. Results may be used to inform doctors and patients about prognosis and advise treatment decisions. For example, risk scores that estimate the 10-year risk of incident cardiovascular disease, like the SCORE2 and QRISK3 [1, 2], may help physicians determine whether initiating primary preventive treatment is appropriate [3–5]. 

Generally, these scores are developed within one cohort, and subsequently externally validated in other cohorts, to assess generalizability and potential suitability for clinical practice [6]. To characterise model performance, several metrics are commonly used (Box 1), among which the Area Under the Curve (AUC), also called “c-statistic”, (range 0.5 to 1; higher is better) is the most prominent [7–9]. The AUC refers to discrimination: a model’s ability to correctly indicate who is more and less likely to have an event. Other commonly reported metrics include the Brier-index (range 0 to 1; lower is better), R^2^ (range 0 to 1; higher is better), and Net Reclassification Improvement (range − 2 to 2; higher is better). These parameters relate to overall model performance (Brier/R^2^) or comparative performance to another score (NRI). Calibration, the agreement between predicted and observed risks across the prediction score range, is predominantly assessed using visual inspection of calibration plots and by comparing observed versus predicted incidence rates [8–11]. 

All these conventional performance metrics have a fixed range, but their interpretation is complex and varies between studies [7–11]. For example, researchers generally consider AUC values under 0.6 unequivocally as ‘poor’, and values above 0.8 as ‘good’, but values between 0.6 and 0.8 may be labelled either as poor, moderate, fair, or good, without any clear rationale for these judgements [7]. These wavering qualifications suggest room for improvement whenever a prediction model’s performance score falls short of perfection. Additionally, validation studies often find that prediction models have worse performance scores in (subgroups of) the validation cohort than in the original development cohort, enduringly fostering appeals for new and improved prediction models [1, 12, 13]. However, prediction models generally do not achieve optimum scores. Estimated event risks between 0 and 100% are inherently inaccurate on the individual level where events either occur or not, and model performance is influenced by population characteristics like the risk score’s distribution. Therefore, although the theoretical optimum of these performance metrics is known, it is unclear what values to realistically expect.

This study aims to demonstrate how a completely accurate, theoretically “perfect”, risk score would perform according to conventionally used performance metrics, assessing the potential effects of population characteristics like score distribution.

Box 1. Performance metrics and interpretation [7–9]

**Table:** 

Metric	Aspect	Description	Interpretation
AUC/C-statistic	Discrimination	Chance that in a random pair of individuals with and without an event, the individual with the event has a higher score. Equates to area under the curve from ROC curve.	Range: 0–1, higher better. Qualifications differ, no clear rationale: − 0.50–0.60: Poor − 0.60–0.75: Poor/moderate/fair − 0.75–0.90: Moderate/fair/good − 0.90-1.00: Good/excellent
Brier Index	Overall performance: captures calibration and discrimination aspects.	Mean of the squared difference between binary outcome Y and predicted risk P (Y-P)^2^ per individual. Maximum (i.e. worst) score depends on incidence. The maximum score for an incidence I is I*(1-I)^2^+(1-I)*I^2^	Range: 1 − 0, lower better. No set value for ‘good’ score. Exponential change from: − 0.00:Perfect − 0.25:Uninformative (if outcome incidence is 50%) Uninformative score decreases with lower outcome incidence E.g. for an overall incidence of 10% the uninformative score is: 0.1*(0.9)^2^ + (0.9)*0.1^2^ = 0.09
Scaled Brier Index	Overall performance: captures calibration and discrimination aspects.	Brier Index score adjusted for uninformative score change with incidence. Expressed as percentage of range from uninformative to perfect: 1-{Brier/[mean(p)*(1-mean(p)]}	Range: 0-100%, higher better. No set value for ‘good’ score. Exponential change from: − 0%:Uninformative − 100%: Perfect Similar to Pearson’s R^2^
R^2^	Overall performance: captures calibration and discrimination aspects.	Variation in outcome explained by predictor. For binary outcomes, Nagelkerke’s R^2^ logarithmic scoring rule is often used	Range: 0–1, higher better. No set value for ‘good’ score. Exponential change from: − 0%:Uninformative − 100%: Perfect Similar to scaled Brier Index
NRI	Reclassification	Relative measure comparing a new prediction score to a reference prediction score. Sum of net proportion of individuals reclassified more appropriately using the new prediction score in the group with an event and in the group without an event separately.	Range: -2 to 2, higher better. No set value for ‘good’ score. A score of 2 means that for all participants with an event the new score is higher than the reference score, and for all participants without an event, the new score is lower than the reference score. A score of -2 means the opposite. Difficult to interpret: (1) proportion of reclassification in the event and non-event group have equal weights regardless of comparative group sizes; (2) insensitive to size of difference between the new and reference prediction; (3) can be false positive with uninformative new markers
Calibration plot	Calibration	Plot of predicted vs. observed outcome incidence in quantiles (usually deciles) of prediction score, or smoothed regression between prediction score and Y. Most relevant for accuracy of risk stratification models.	Predicted/observed (P/O) outcome proportion should be equal in every decile. The regression line should align with P/O as floating average or in individual deciles. The regression slope should be 1, and the intercept 0. Intercepts higher/lower than zero indicate systematic over/under estimation.

## Methods

**Fig. 1:**
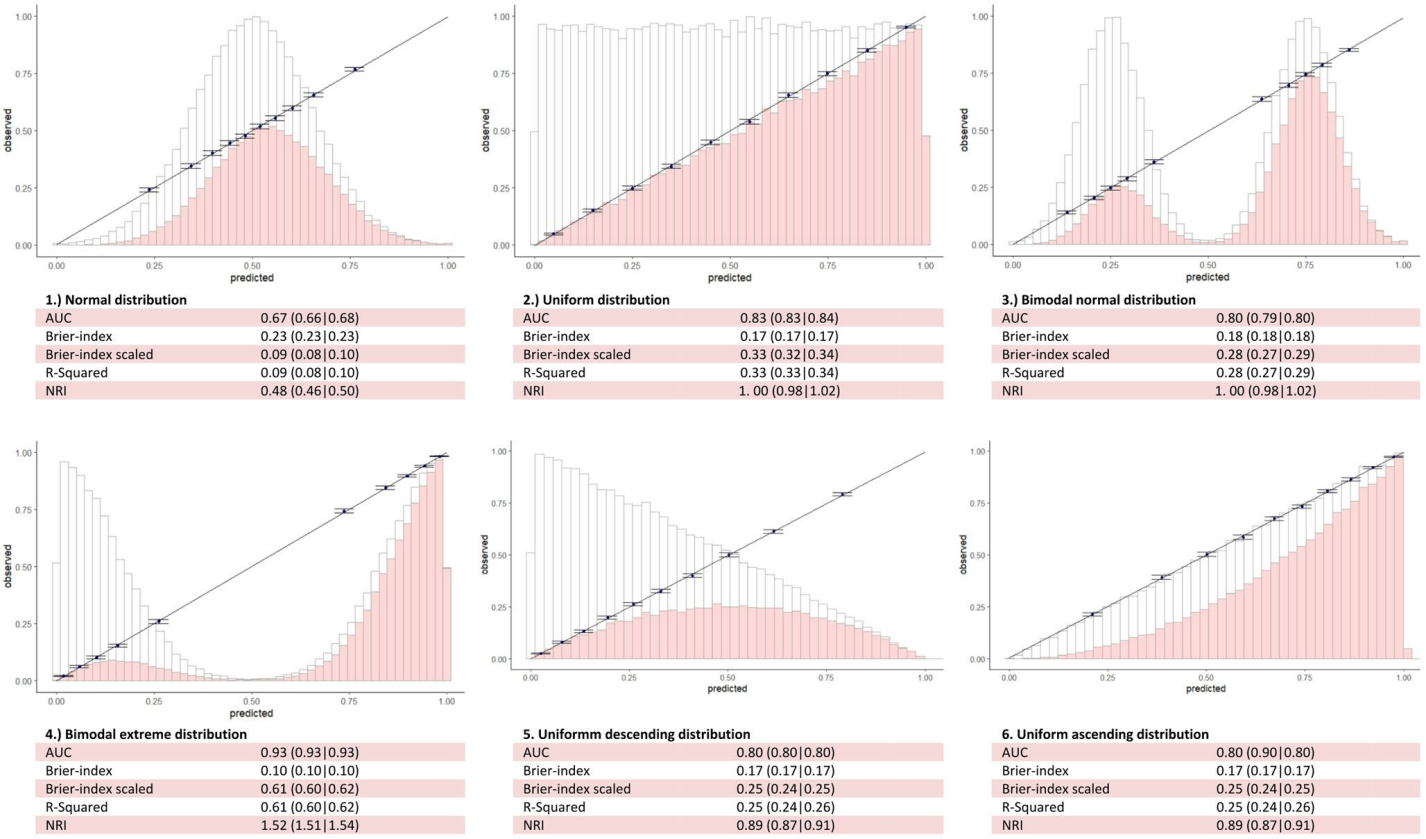
Results for the main analyses per distribution. The figures depict the histograms for the distributions with individuals with an event shaded in pink. Projected on top are the calibration plots between the observed and predicted incidence rates. Below each distribution are the performance results according to each metric, with (minimum|maximum) range based on 1,000 bootstraps. Abbreviations: AUC=Area Under the Curve, NRI = Net Reclassification Improvement

Data were simulated for 100,000 individuals using R version 4.3.1. Individuals were assigned a random risk score denoting their event risk from 0% to 100%, according to six different, hypothetical distributions (Fig. 1; Online Methods 1): (1) normal distribution; (2) uniform distribution with scores equally distributed from zero to one; (3) bimodal distribution; (4) bimodal distribution centred around the two extreme values (0 and 1); (5) left centred distribution; (6) right centred distribution.

An outcome variable equalling 1 (meaning an event, e.g. stroke) or 0 (no event, e.g. no stroke) was then calculated for each distribution that was perfectly compliant with the risk score: individuals with a score of 0.01 had a 1% risk of an event 1 as outcome, individuals with a score of 0.99 had a 99% chance of a 1 as outcome etc. Hereafter, the relation between the risk score and the outcome variable was assessed according to the investigated metrics: the AUC, Brier-index, the scaled Brier-index, the continuous NRI, and R^2^. Since the continuous NRI is a relative metric, comparing a model’s performance to that of a previous model, the mean incidence of the outcome was taken as comparison (comparable to an intercept or the a-priori risk for the population), which is the “net reclassification from the null model” (NRI_0_) as defined by Pencina et al. [14] These simulations were repeated 1,000 times to account for random variability in the simulated distributions. From the results, the median, minimum and maximum value of the bootstraps are reported for each metric. For illustrative purposes, histograms were generated for each of the six distributions, with the corresponding events, and the corresponding calibration plot using the package CalibrationCurves.

Several sensitivity analyses were performed. First, to assess the influence of the risk range, analyses were repeated halving the risk scores, so that scores ranged from 0 to 0.5. Subsequently, the range of the risk score was shifted around the mean value, by halving risk scores as above, but adding 0.25 so that the centre of each distribution remained at 0.5 and the score now ranges from 0.25 to 0.75. This was repeated adding 0.50 so that the centre of each distribution was at 0.75 and scores ranged from 0.5 to 1.0. Second, the influence of the granularity of the risk score was evaluated, by rounding the risk score values to the nearest 10% (i.e. 0.1, 0.2, 0.3 etc.). Third, the influence of manipulating the risk distribution was evaluated, binning the risk score into 10 equally sized groups according to deciles of the risk prediction values, a strategy commonly used to categorize variables. Finally, an analysis was performed to evaluate the performance metric values if all individuals below and above a specific risk score threshold would have an outcome of ‘no event’ and ‘event’ respectively. This was done for random thresholds between 0 and 1 for each of the 1,000 bootstraps, thus assessing the range of possible performance values for all thresholds between 0 and 1.

To let readers assess other common distributions not included in this paper, like Weibull and Gamma distributions, a shiny app was created (https://jan-willem-van-dalen.shinyapps.io/app-1/) where users can define their own distributions and their corresponding analysis results.

Finally, to assess how the net benefit is affected by these score distributions, decision curve analysis graphs were constructed. These graphs depict the net benefit (plotted on the y-axis) of treating participants above a predicted risk level (plotted on the x-axis) compared to treating everyone and treating no one [15]. The “net benefit” is recommended above methods like the NRI to evaluate clinical utility. A model is generally considered clinically useful if its net benefit is higher than both ‘treat all’ and ‘treat none’ across a range of plausible risk thresholds. The clinical value of the model is greatest at the threshold where the gap between the model’s net benefit and the next best strategy is widest.

## Results

Figure 1 presents the results for the main analyses. Depicted are the histograms for each score distribution with individuals with an event shaded in red, and the calibration plot for the analyses projected overhead. Below each figure are the prediction metric values for the corresponding distribution. For a normally distributed risk score with perfect accuracy, the AUC was 0.67, the Brier-Index (BI) 0.23, the Brier-index scaled (BS) 0.08, the R^2^ 8%, and the NRI 0.48. These values were better for a uniform (AUC = 0.83, BI = 0.17, BS = 0.33, R^2^ = 33%, NRI = 1.0) and bimodal normal distribution (AUC = 0.80, BI = 0.18, BS = 0.28, R^2^ = 28%, NRI = 1.00). Prediction metric values were best for the extreme bimodal distribution (AUC = 0.93, BI = 0.10, BS = 0.61, R^2^ = 61%, NRI = 1.52). Values for a uniform descending and uniform ascending distribution were identical (both: AUC = 0.80, BI = 0.17, BS = 0.25, R^2^ = 25%, NRI = 0.89). All these estimates had narrow bootstrap ranges. For all these distributions, the calibration plot perfectly lined up the observed and predicted rates, with slopes of 1 and intercepts of 0, confirming the risk score’s perfect accuracy. Box 2 provides an example of a real-world clinical interpretation of this.

Results of the sensitivity analyses are summarized in Table 1. Supplementary Figs. 1–6 depict the effect of the sensitivity analyses on the histograms and calibration plots for the different distributions. When the range of risk scores was narrowed, AUC, BS, R^2^ and NRI values were lower. The Brier-Index showed similar patterns, except that for the normal and the asymmetrical distributions values in narrower ranges were lower than for the original analyses. For all ranges, calibration was perfect according to the calibration plots, with an intercept of 0 and a slope of 1 (Supplementary Figs. 1–6, panels 1–3). For the sensitivity analysis evaluating rounded risk scores as predictors, results were practically identical to those for the original analyses, with perfect calibration according to the calibration plots (Supplementary Figs. 1–6, panel 4). For the sensitivity analyses categorizing the prediction score into deciles, results were also identical to the original scores for all metrics, except the NRI, which was 0 for each distribution. In these analyses, the calibration plots deviated between the predicted and observed risks, with calibration slopes diverging from 1 and intercepts other than 0 (Supplementary Figs. 1–6, panel 5). For the analyses dichotomising outcomes based on risk score thresholds from 0 to 1, perfect values of 1, 0, and 1 respectively were consistently observed for the AUC, Brier-index and BS for all the evaluated distributions (Supplementary Fig. 7). This is the only analysis in which the BS differed notably from the R^2^ values. Values for the R^2^ and NRI varied depending on the threshold chosen (hence the min-max practically spanning the full range of the scores). For these analyses, the calibration plots showed great differences between predicted and observed risks, with extreme divergence of the calibration intercept and slope from 0 to 1 respectively. Finally, the decision curve analyses graphs (Supplementary Fig. 8) show that the net benefit also varies across the different risk distributions. However, all graphs demonstrate that the prediction model exceeds the alternative strategies (treat all/treat none). The greatest clinical value of the model, according to the difference between the prediction model and the next best strategy, is consistently positioned at the mean population risk.

**Table 1:** Results of the original main analyses and sensitivity analyses for the analysed performance metrics

Metric	Distribution	Original		Score range		Rounded score	Deciles	Random
		analyses	0.00-0.50	0.25–0.75	0.50-1.00			threshold
AUC	1. Normal	0.67 (0.66|0.68)	0.61 (0.61|0.62)	0.58 (0.58|0.59)	0.61 (0.61|0.62)	0.66 (0.66|0.67)	0.67 (0.66|0.67)	1 (1|1)
	2. Uniform	0.83 (0.83|0.84)	0.72 (0.72|0.73)	0.72 (0.72|0.73)	0.72 (0.72|0.73)	0.83 (0.83|0.83)	0.83 (0.83|0.83)	1 (1|1)
	3. Bimodal normal	0.80 (0.79|0.80)	0.70 (0.69|0.70)	0.65 (0.64|0.65)	0.70 (0.69|0.70)	0.79 (0.79|0.80)	0.79 (0.79|0.80)	1 (1|1)
	4. Bimodal extreme	0.93 (0.93|0.93)	0.79 (0.78|0.79)	0.72 (0.71|0.72)	0.79 (0.78|0.79)	0.93 (0.92|0.93)	0.93 (0.93|0.92)	1 (1|1)
	5. Uniform descending	0.80 (0.80|0.80)	0.74 (0.74|0.74)	0.64 (0.63|0.64)	0.65 (0.64|0.66)	0.80 (0.79|0.80)	0.80 (0.79|0.80)	1 (1|1)
	6. Uniform ascending	0.80 (0.80|0.80)	0.65 (0.65|0.65)	0.64 (0.63|0.64)	0.74 (0.74|0.74)	0.80 (0.79|0.80)	0.80 (0.79|0.80)	1 (1|1)
Brier-index	1. Normal	0.23 (0.23|0.23)	0.18 (0.18|0.18)	0.24 (0.24|0.25)	0.18 (0.18|0.18)	0.23 (0.23|0.23)	0.23 (0.23|0.23)	0 (0|0)
	2. Uniform	0.17 (0.17|0.17)	0.17 (0.17|0.17)	0.17 (0.17|0.17)	0.17 (0.17|0.17)	0.17 (0.17|0.17)	0.17 (0.17|0.17)	0 (0|0)
	3. Bimodal normal	0.18 (0.18|0.18)	0.17 (0.17|0.17)	0.23 (0.23|0.23)	0.17 (0.17|0.17)	0.18 (0.18|0.18)	0.18 (0.18|0.19)	0 (0|0)
	4. Bimodal extreme	0.10 (0.10|0.10)	0.15 (0.15|0.15)	0.21 (0.21|0.21)	0.15 (0.15|0.15)	0.10 (0.10|0.10)	0.10 (0.10|0.10)	0 (0|0)
	5. Uniform descending	0.17 (0.17|0.17)	0.13 (0.12|0.13)	0.23 (0.23|0.23)	0.21 (0.21|0.21)	0.17 (0.17|0.17)	0.17 (0.17|0.17)	0 (0|0)
	6. Uniform ascending	0.17 (0.17|0.17)	0.21 (0.21|0.21)	0.23 (0.23|0.23)	0.13 (0.12|0.13)	0.17 (0.17|0.17)	0.17 (0.17|0.17)	0 (0|0)
Brier scaled	1. Normal	0.09 (0.08|0.10)	0.03 (0.03|0.03)	0.02 (0.02|0.03)	0.03 (0.03|0.03)	0.09 (0.08|0.09)	0.08 (0.08|0.09)	1.00 (1.00|1.00)
	2. Uniform	0.33 (0.32|0.34)	0.11 (0.10|0.11)	0.08 (0.08|0.09)	0.11 (0.10|0.11)	0.33 (0.32|0.34)	0.33 (0.32|0.33)	1.00 (1.00|1.00)
	3. Bimodal normal	0.28 (0.27|0.29)	0.09 (0.09|0.10)	0.07 (0.07|0.07)	0.09 (0.09|0.10)	0.27 (0.26|0.28)	0.26 (0.26|0.27)	1.00 (1.00|1.00)
	4. Bimodal extreme	0.61 (0.60|0.62)	0.20 (0.19|0.21)	0.15 (0.15|0.16)	0.02 (0.19|0.21)	0.61 (0.60|0.61)	0.59 (0.58|0.60)	1.00 (1.00|1.00)
	5. Uniform descending	0.25 (0.24|0.25)	0.09 (0.087|0.1)	0.06 (0.05|0.06)	0.06 (0.06|0.07)	0.24 (0.24|0.25)	0.24 (0.24|0.25)	1.00 (0.98|1.00)
	6. Uniform ascending	0.25 (0.24|0.25)	0.06 (0.06|0.07)	0.06 (0.05|0.06)	0.09 (0.09|0.10)	0.24 (0.24|0.25)	0.24 (0.23|0.25)	1.00 (0.99|1.00)
R-squared	1. Normal	0.09 (0.08|0.10)	0.03 (0.03|0.03)	0.02 (0.02|0.03)	0.03 (0.03|0.03)	0.09 (0.08|0.09)	0.08 (0.08|0.09)	0.25 (0.01|0.64)
	2. Uniform	0.33 (0.32|0.34)	0.11 (0.11|0.12)	0.11 (0.11|0.12)	0.11 (0.11|0.12)	0.33 (0.32|0.34)	0.33 (0.32|0.34)	0.59 (0.06|0.75)
	3. Bimodal normal	0.28 (0.27|0.29)	0.09 (0.09|0.10)	0.07 (0.07|0.07)	0.09 (0.09|0.10)	0.27 (0.27|0.28)	0.26 (0.25|0.27)	0.56 (0.00|0.91)
	4. Bimodal extreme	0.61 (0.60|0.62)	0.20 (0.20|0.21)	0.15 (0.15|0.16)	0.20 (0.20|0.21)	0.61 (0.60|0.62)	0.55 (0.55|0.56)	0.89 (0.09|0.95)
	5. Uniform descending	0.25 (0.24|0.26)	0.10 (0.10|0.11)	0.06 (0.05|0.06)	0.06 (0.06|0.07)	0.25 (0.24|0.25)	0.24 (0.23|0.24)	0.53 (0.00|0.72)
	6. Uniform ascending	0.25 (0.24|0.26)	0.06 (0.06|0.07)	0.06 (0.05|0.06)	0.10 (0.10|0.10)	0.25 (0.24|0.25)	0.24 (0.23|0.24)	0.52 (0.01|0.72)
NRI	1. Normal	0.48 (0.46|0.50)	0.32 (0.30|0.34)	0.24 (0.22|0.25)	0.32 (0.31|0.34)	0.45 (0.44|0.47)	0 (0|0)	0.00 (0.00|1.66)
	2. Uniform	1. 00 (0.98|1.02)	0.67 (0.65|0.69)	0.67 (0.65|0.68)	0.67 (0.65|0.68)	0.99 (0.98|1.00)	0 (0|0)	0.74 (0.04|1.94)
	3. Bimodal normal	1. 00 (0.98|1.02)	0.67 (0.65|0.68)	0.50 (0.48|0.52)	0.67 (0.65|0.68)	1.00 (0.99|1.02)	0 (0|0)	1.03 (0.00|2.00)
	4. Bimodal extreme	1.52 (1.51|1.54)	1.01 (1.00|1.03)	0.76 (0.74|0.78)	1.01 (1.00|1.03)	1.52 (1.51|1.53)	0 (0|0)	1.86 (0.31|2.00)
	5. Uniform descending	0.89 (0.87|0.91)	0.71 (0.69|0.73)	0.41 (0.39|0.43)	0.45 (0.43|0.46)	0.89 (0.87|0.90)	0 (0|0)	0.46 (0.00|1.94)
	6. Uniform ascending	0.89 (0.87|0.91)	0.45 (0.43|0.73)	0.41 (0.39|0.43)	0.71 (0.69|0.46)	0.89 (0.87|0.90)	0 (0|0)	0.46 (0.00|1.94)

Depicted are the results with (minimum|maximum) range for each metrics per sensitivity analyses, with the results of the original main analyses in the most left column for reference. For the ‘Score range’ analyses, the influence of the score range was assessed, by adapting the range of each distribution to span 0.00-0.50, 0.25–0.75, and 0.50-1.00 respectively. For the ‘Rounded score’ analyses, the influence of the score’s granularity was assessed, by rounding the risk scores to the nearest 0.1 after the events had been generated. For the “Deciles” analysis, individuals were categorized in 10 equal sized groups according to deciles of their risk score. For the “Random cut-off” analyses, for each iteration of the bootstrap, a random threshold was chosen above which all individuals experienced an outcome event. The figures corresponding to these analyses are depicted in Supplementary Tables 1 through 6. Abbreviations: AUC=Area Under the Curve, NRI = Net Reclassification Improvement

Box 2. Real world example

**Table:** 

*Imagine a hypothetical patient*,* with a risk of incident cardiovascular disease that is a-priori normally distributed across the population. A physician using a risk prediction tool with perfect calibration -meaning that from every 100 patients with a 25% risk*,* 25 get a cardiovascular event- can expect a maximum AUC of 0.67 for that prediction tool. Prediction tools with higher AUC are increasingly inaccurate: if the AUC would be 1*,* either none or all the 100 patients with a 25% risk would get an event*,* and the calibration measures of the prediction tool would be very poor. Other commonly used measures like the Brier-score*,* the explained variation (R*^*2*^*) and NRI are similarly uninformative for this example. Therefore*,* the performance of such a risk estimating prediction tool for clinical implementation should be judged by calibration measures.*

## Discussion

The analyses showed that even with a fully accurate, “perfect” risk score, performance values on the AUC, Brier-Index, R^2^ and NRI vary widely depending on the risk score’s distribution. For a normally distributed score, performance indices will never attain values that exceed characterization as ‘poor’ to ‘moderate’. The best performance values occurred when an extreme bimodal distribution was hypothesized. Under other distributions, values did not exceed performance usually considered ‘moderate’ to ‘good’. Narrowing the range of the distributions worsened performance values. Reducing risk score granularity reduced performance values only marginally. Binning into deciles generally worsened performance values only slightly, except for the NRI, which lost all improvement over the a-priori population risk. As expected based on how data were simulated, calibration plots were perfect for all analyses above except the binned analysis where the observed number of events diverged from what would be expected. Dichotomising outcomes based on thresholds between 0 and 1 gave perfect AUC and Brier values, values varying across the range of the NRI and R^2^ depending on the distribution and threshold, and calibration plots completely deviating from perfect.

The current study clearly shows that prediction model performance according to several commonly used performance metrics depend strongly on the risk distribution. The range of performance scores for specific distributions that may be commonly encountered in practice illustrate what might be realistically expected for different metrics in validation studies. Crucially, this study underlines the importance of using the right metric for the right research question. A few studies have previously warned about the effects that distribution may have on the AUC [16–18]. The AUC is optimally suited for tests meant to dichotomize individuals into yes or no event with full accuracy, e.g. a blood test to diagnose acute myocardial infarction [19], effectively providing a summary estimate of a test’s sensitivity and specificity. However, the AUC is relatively uninformative to judge how accurately a population risk prediction score like the SCORE or QRISK3 captures the risk percentages for individuals, although clinically useful scores may have high AUCs [20]. The AUC is also insensitive to the added value of additional covariates that are strongly associated with increased risk but do not increase the bimodality of the score distribution, which may render AUC differences uninformative for evaluating the added value of an updated model [16, 21]. Although the Brier-Index is mostly a measure of overall model performance, regarded to relate to both discrimination and calibration [8], the current analyses show that values stay relatively close to the uninformative value of 0.25 for any other than the most extreme dichotomized risk score distributions. R^2^ and NRI scores suffer the same downsides but also lack a clear interpretation [8, 9, 22]. The NRI has been additionally criticized, mostly for making uninformative new markers to appear predictive, and its use has therefore been advised against altogether [15, 23]. 

These results have important implications for studies developing and validating risk prediction scores. For prediction tools aiming to categorize populations into two groups, for example when evaluating diagnostic tests, measures like the AUC and Brier-Index may be good performance indicators. But for scores that assign a certain risk percentage to individuals for developing events in a specific time window, calibration may be the most valuable aspect to assess the accuracy of those predictions for individual patients when scores are applied in clinical practise. This has been argued previously, but seemingly with limited uptake [16]. Traditionally, studies seem to focus on the AUC, and assessment of calibration performance of risk prediction models receives little attention [10]. A search of PubMed for papers validating two recent prominent risk prediction scores used for stratification in cardiovascular disease: the SCORE2 and QRISK3 (Supplementary Table 1) identified 44 identified abstracts [1, 2, 12, 13, 24–64], 96% of which reported the AUC (41% as the main focus) while only 22% reported numbers regarding calibration (slopes or predicted/observed), 67% rated calibration qualitatively, and 26% did not report calibration at all. Papers that developed new or adapted scores generally expressed the benefit of the new score as difference in AUC [39, 42–46, 51, 56] e.g. reporting that the c-index improved by 0.03. This clearly illustrates the emphasis placed on the AUC over calibration statistics, even in risk stratification scores. The AUC’s prominence and its popular qualitative labels as poor, moderate, good, or excellent may be unhelpful. Studies may report a range of “excellent” AUCs of > 0.85 and mention “satisfactory calibration” which is a highly ambiguous term [39, 40]. Accurately reporting calibration is important because poorly calibrated predictions can be misleading and result in incorrect and possibly harmful treatment decisions [10]. Vice versa, a perfectly calibrated score of a risk that is normally distributed in the population, will never score higher than 0.67 on the AUC, receiving the label “poor”, while the score is in fact perfectly accurate for assessing an individual patient’s risk percentage. Developing better scores with higher AUCs may seem desirable, but if these scores achieve higher AUCs at the cost of worse calibration, they may end up being less suited for their clinical purpose: correctly estimate the risk percentage of a future event for an individual patient [17]. For claims on making better decisions, dedicated measures like the Net Benefit and its associated decision curve may be helpful [21]. However, although this relates to the clinical usefulness compared to a previous decision strategy, it does not capture calibration.

The dichotomised analyses highlight the importance of understanding the distribution of both the risk score and the events. For example, a 10-year mortality risk score incorporating age will likely have excellent AUC and Brier values when evaluated in a population comprising younger and older people, because of the threshold effect of mortality rising sharply in old-age: young people have a very low risk, old people form a certain age a very high risk. Such a score may be labelled excellent in a population aged from 19 to 80, but poor in one aged 70–80 [1, 12, 13]. The R^2^ may inform on the general model fit, but is also sensitive to distribution effects, complicating direct comparison between scores in different populations. The NRI suffers from the same distribution problems and has no clear interpretation, so may be the least informative metric. Understanding the risk score distribution in the population and the evaluation of calibration is again paramount to judge these scores.

Evaluating, interpreting, and reporting calibration may be complicated by the relative lack of widely accepted and understood quantification metrics. Calibration plots rely on visual inspection, inviting subjective interpretation and qualitative descriptions. Some researchers use the Hosmer-Lemshow test, which has many drawbacks: it artificially groups participants in risk strata, yields a p-value uninformative of the type and extent of miscalibration, and has low statistical power [10, 11]. Calibration may also be quantified by the ratio of predicted versus observed incidence rates. These have the downside that predicted/observed rates for the whole score may not reflect rates in the score’s subregions that bear clinical relevance for individual patients, while regional over- and underprediction balance out overall. For example, scores may overestimate risks above 70%, and underestimate risks below 30%, but score perfectly on average. This can be problematic if specific ranges are used for making treatment decisions, for example when patients only qualify for preventive treatment when an event risk is > 20%. Reporting calibration intercept and slope with 95% confidence intervals is a better alternative. Finally, flexible calibration curves allow for capturing deviation at different regions of the score range but require relatively large datasets and lack a clear numerical summary measure. Research efforts into developing ways to accurately capture calibration in a small set of easily interpretable numbers, and an unequivocal consensus statement on how calibration is to be reported may help researchers to focus on calibration. Until then it is advisable for evaluation of risk prediction scores to report calibration slopes and intercepts, and regional over- and underprediction [10]. Also, it may be recommendable to report distribution of risk prediction scores in the population, and the corresponding distribution of the event rates.

A limitation of this study is that many more, different distributions could be envisioned, but the current results should give a good sense of how important distribution is for prediction performance metrics, and what types of scores are generally to be expected with which types of distributions. To compensate, this study developed a shiny app (https://jan-willem-van-dalen.shinyapps.io/app-1/) where users can define their own distribution parameters (including for Gamma, Weibull and completely custom distributions) to assess the corresponding prediction score results. Another limitation is that the operationalisation of perfectly accurate risk score assumes the underlying risk ranges between 0% and 100%, while an actual “perfect” risk score would only have 0% and 100% scores. It may be argued that any estimation between 0% and 100% is suitable for improvement, since an event either occurs or not. However, this suggests that incident disease over many years (e.g. 10 years for SCORE and QRISK3) is fully predictable given all the relevant parameters and not a stochastic process. However, this divide may occur in diagnostic testing or with autosomal dominant conditions that inevitably and exclusively result in a future phenotype but is very unlikely to occur in multifactorial diseases like cardiovascular disease or dementia. Finally, it must be noted that the continuous NRI as operationalised in this paper may not correspond with the model calibration, but when the estimated risk is categorized based on cut-points, the cut-points introduce at least some aspect of calibration [65]. However, given that the analyses based on deciles of the a-priori risk yielded similarly poor NRI values suggests that this mostly occurs when categorized into a smaller number of groups (e.g. tertiles, quartiles).

## Conclusion

Commonly used model performance measures like the AUC, Brier-index, R^2^, and NRI, depend strongly on risk distributions. They may not exceed values generally considered poor/moderate in many common distributions, and only exceed moderate/good values if the risk is divided between two extremes in the distribution. Therefore, the performance of prediction scores for evaluating an individuals’ risk percentage should focus less on these metrics and more on calibration to determine whether a score accurately predicts risk. When reporting results of risk prediction score validation, researchers should preferably report risk score distributions and equally emphasize calibration metrics like regional observed vs. predicted incidence rates, calibration intercepts and slopes next to discrimination metrics. Most importantly, clinicians looking to accurately estimate event risks to inform individual patients and make treatment decisions in the consultation room, should make sure that the utilized prediction tools have excellent calibration.

## Supplementary Information

Below is the link to the electronic supplementary material.

Supplementary Material 1
