# Supplementary material for: Risk prediction for individual patients and the pitfalls of selecting an optimal prediction model: do not judge a model by its c-statistic
Source: Eur J Epidemiol. 2026 May 27;41(4):395–404. doi: 10.1007/s10654-026-01378-2 (PMC13331923; doi:10.1007/s10654-026-01378-2)
Supplement: Supplementary file 1 — Supplementary Material 1 [file 10654_2026_1378_MOESM1_ESM.pdf]

Online Supplement to

“Risk prediction for individual patients and the pitfalls of selecting an optimal prediction model”

Contents **Page**

Online Methods 1. Details on risk distributions 2

Supplementary Figure 1. Results for the sensitivity analyses for the normal distribution. 3

Supplementary Figure 2. Results for the sensitivity analyses for the uniform distribution. 4

Supplementary Figure 3. Results for the sensitivity analyses for the bimodal normal distribution. 5

Supplementary Figure 4. Results for the sensitivity analyses for the bimodal extreme distribution. 6

Supplementary Figure 5. Results for the sensitivity analyses for the uniform descending distribution. 7

Supplementary Figure 6. Results for the sensitivity analyses for the uniform ascending distribution. 8

Supplementary Figure 7. Performance metric values for the different distributions according to the threshold above which everyone experiences an event. 9

Supplementary Table 1. Mentions of AUC and calibration related performance in abstracts of validation studies for the QRISK3 and SCORE2 cardiovascular risk scores. 10

Online Methods 1. Details on risk distributions:

Individuals were assigned a random risk score denoting their event risk from 0% to 100%, according to six different distributions: 1.) A normal distribution with a mean of 0.5 and a standard deviation of (0.5/3.3)=0.15 so that the distribution spanned the whole range between zero and one and 99.9% of the observations were within this range; 2.) A uniform distribution with scores equally distributed from zero to one; 3.) A bimodal distribution with half of scores normally distributed around 0.25 and the other half round 0.75, both with an SD of (0.25/3.3)=0.08 so that values spanned the whole range between zero and one with 99.9% of observations within this range; 4.) A bimodal distribution centred around the two extreme values of zero and one, which was attained by taking a normal distribution with a mean of 0 and a standard deviation of 0.15, and adding 1 to all values under zero; 5.) A left centred distribution with 2% of individuals having risk scores approximating 0%, then linearly declining (1.98% having scores around 1%, 1.96% having scores around 2%, 1.94% having scores around 3% etc.) to nearly 0% of individuals with risk scores approximating 100%; 6.) A right centred distribution with 2% of individuals having risk scores approximating 100%, then linearly declining to nearly no individuals with risk scores approximating 0%.

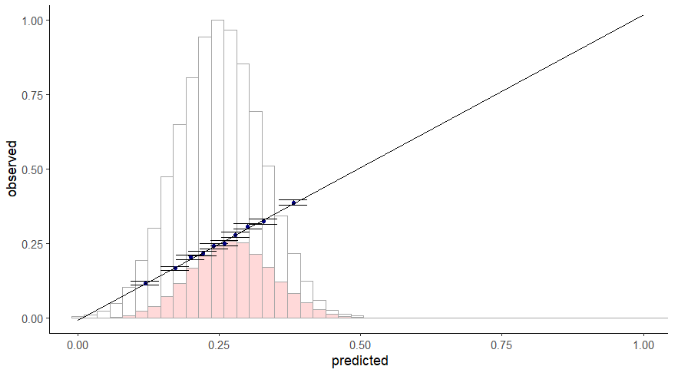

1.) Sensitivity analysis: score range 0.00-0.50

AUC	0.61 (0.61|0.62)
Brier-index	0.18 (0.18|0.18)
Brier-index scaled	0.03 (0.03|0.03)
R-Squared	0.03 (0.03|0.03)
NRI	0.32 (0.30|0.34)

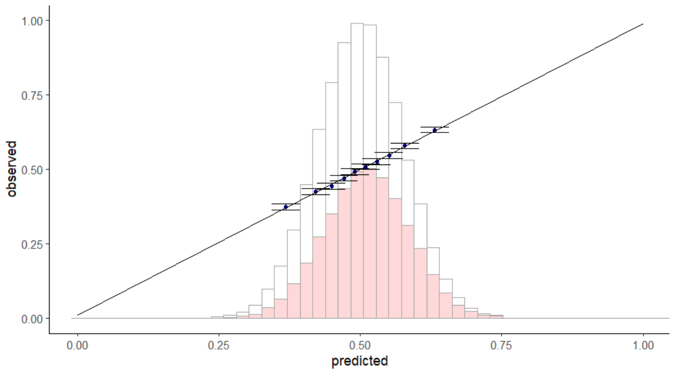

2.) Sensitivity analysis: score range 0.25-0.75

AUC	0.58 (0.58|0.59)
Brier-index	0.24 (0.24|0.25)
Brier-index scaled	0.02 (0.02|0.03)
R-Squared	0.02 (0.02|0.03)
NRI	0.24 (0.22|0.25)

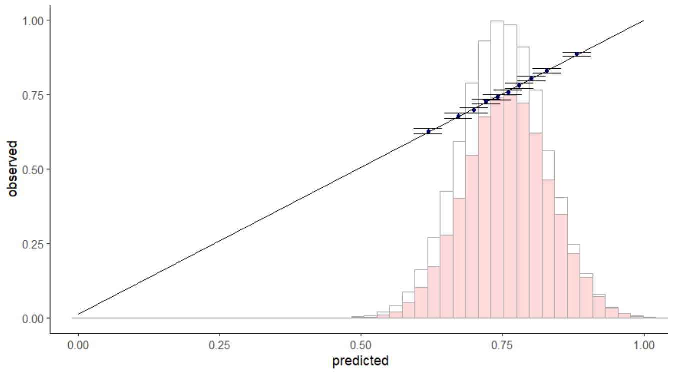

3.) Sensitivity analysis: score range 0.50-1.00

AUC	0.61 (0.61|0.62)
Brier-index	0.18 (0.18|0.18)
Brier-index scaled	0.03 (0.03|0.03)
R-Squared	0.03 (0.03|0.03)
NRI	0.32 (0.31|0.34)

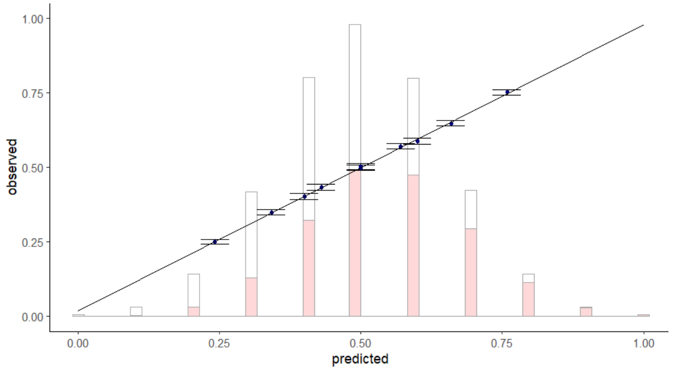

4.) Rounded score

AUC	0.66 (0.66|0.67)
Brier-index	0.23 (0.23|0.23)
Brier-index scaled	0.09 (0.08|0.09)
R-Squared	0.09 (0.08|0.09)
NRI	0.45 (0.44|0.47)

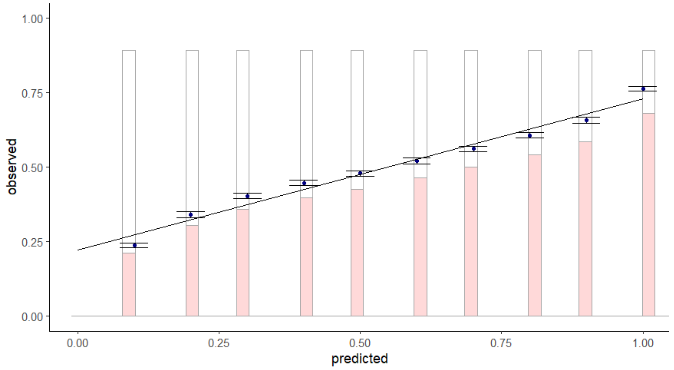

5.) Deciles

AUC	0.67 (0.66|0.67)
Brier-index	0.23 (0.23|0.23)
Brier-index scaled	0.08 (0.08|0.09)
R-Squared	0.08 (0.08|0.09)
NRI	0 (0|0)

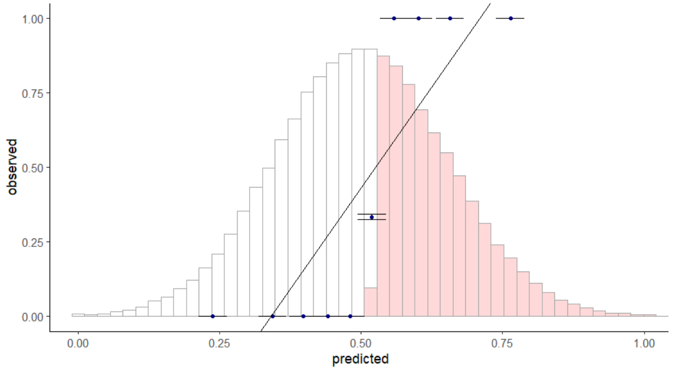

6.) Random threshold

AUC	1 (1|1)
Brier-index	0 (0|0)
Brier-index scaled	1.00 (1.00|1.00)
R-Squared	0.25 (0.01|0.64)
NRI	0.00 (0.00|1.66)

Supplementary Figure 1. Results for the sensitivity analyses for the normal distribution. The figures depict the histograms for the normal distribution across the sensitivity analyses with individuals with an event shaded in pink. Projected on top are the calibration plots between the observed and predicted incidence rates. Below each distribution are the performance results for each metric, with (minimum|maximum) range based on 1,000 bootstraps. For the 'Score range' analyses, the influence of the score range was assessed, by adapting the range of each distribution to span 0.00-0.50, 0.25-0.75, and 0.50-1.00 respectively. For the 'Rounded score' analyses, the influence of the score's granularity was assessed, by rounding the risk scores to the nearest 0.1 after the events had been generated. For the "Deciles" analysis, individuals were categorized in 10 equal sized groups according to deciles of their risk score. For the "Random cut-off" analyses, for each iteration of the bootstrap, a random threshold was chosen above which all individuals experienced an outcome event. Abbreviations: AUC=Area Under the Curve, NRI=Net Reclassification Improvement

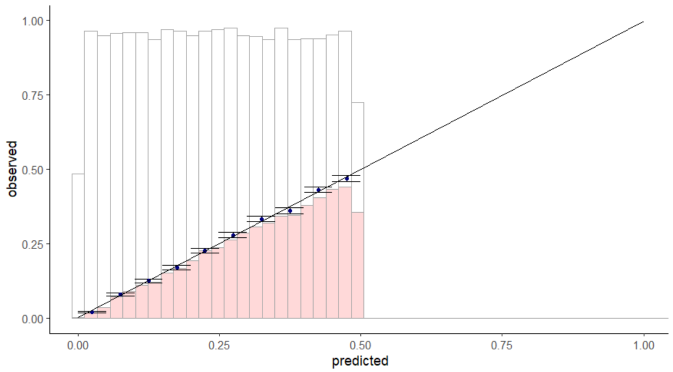

1.) Sensitivity analysis: score range 0.00-0.50

AUC	0.72 (0.72|0.73)
Brier-index	0.17 (0.17|0.17)
Brier-index scaled	0.33 (0.32|0.34)
R-Squared	0.11 (0.11|0.12)
NRI	0.67 (0.65|0.69)

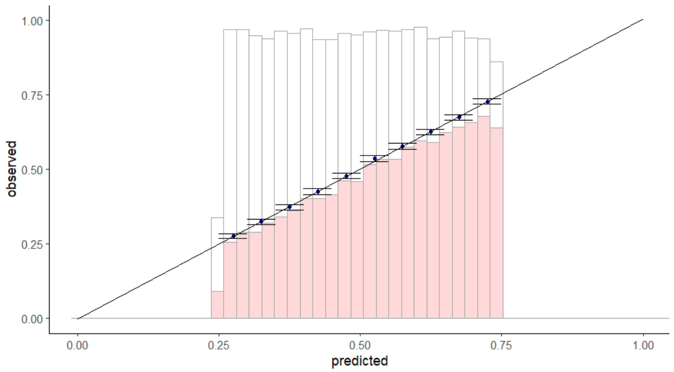

2.) Sensitivity analysis: score range 0.25-0.75

AUC	0.72 (0.72|0.73)
Brier-index	0.17 (0.17|0.17)
Brier-index scaled	0.11 (0.10|0.11)
R-Squared	0.11 (0.11|0.12)
NRI	0.67 (0.65|0.68)

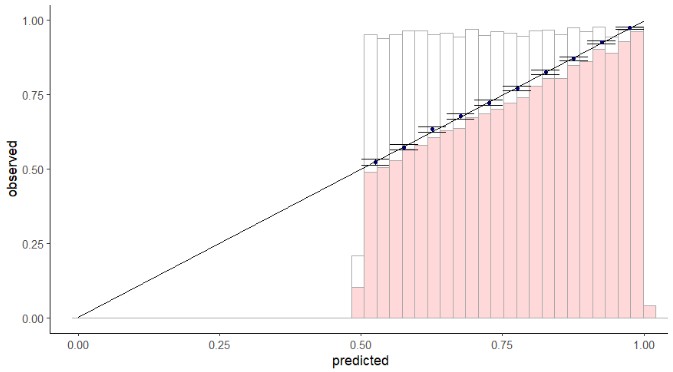

3.) Sensitivity analysis: score range 0.50-1.00

AUC	0.72 (0.72|0.73)
Brier-index	0.17 (0.17|0.17)
Brier-index scaled	0.11 (0.10|0.11)
R-Squared	0.11 (0.11|0.12)
NRI	0.67 (0.65|0.68)

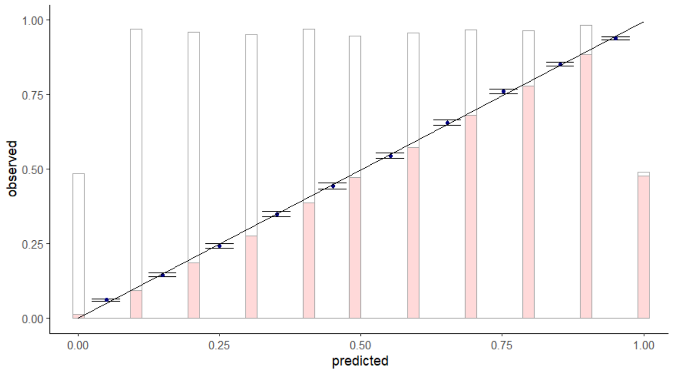

4.) Rounded score

AUC	0.83 (0.83|0.83)
Brier-index	0.17 (0.17|0.17)
Brier-index scaled	0.33 (0.32|0.34)
R-Squared	0.33 (0.32|0.34)
NRI	0.99 (0.98|1.00)

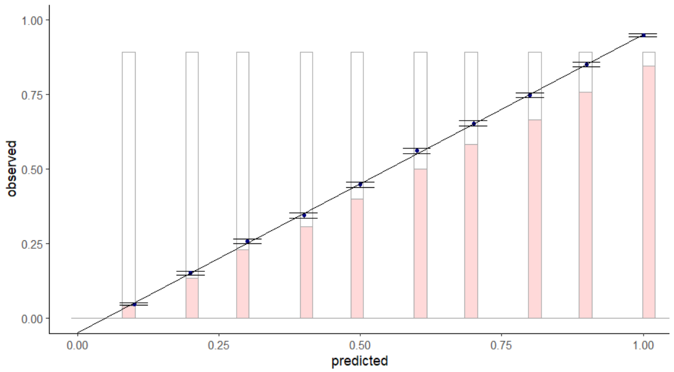

5.) Deciles

AUC	0.83 (0.83|0.83)
Brier-index	0.17 (0.17|0.17)
Brier-index scaled	0.33 (0.32|0.33)
R-Squared	0.33 (0.32|0.34)
NRI	0 (0|0)

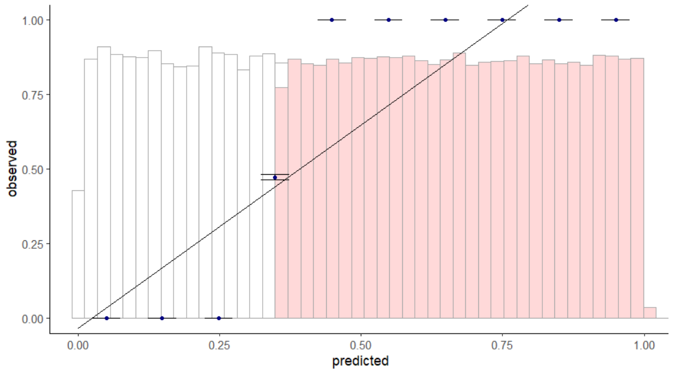

6.) Random threshold

AUC	1 (1|1)
Brier-index	0 (0|0)
Brier-index scaled	1.00 (1.00|1.00)
R-Squared	0.59 (0.06|0.75)
NRI	0.74 (0.04|1.94)

Supplementary Figure 2. Results for the sensitivity analyses for the uniform distribution. The figures depict the histograms for the uniform distribution across the sensitivity analyses with individuals with an event shaded in pink. Projected on top are the calibration plots between the observed and predicted incidence rates. Below each distribution are the performance results for each metric, with (minimum|maximum) range based on 1,000 bootstraps. For the 'Score range' analyses, the influence of the score range was assessed, by adapting the range of each distribution to span 0.00-0.50, 0.25-0.75, and 0.50-1.00 respectively. For the 'Rounded score' analyses, the influence of the score's granularity was assessed, by rounding the risk scores to the nearest 0.1 after the events had been generated. For the "Deciles" analysis, individuals were categorized in 10 equal sized groups according to deciles of their risk score. For the "Random cut-off" analyses, for each iteration of the bootstrap, a random threshold was chosen above which all individuals experienced an outcome event. Abbreviations: AUC=Area Under the Curve, NRI=Net Reclassification Improvement

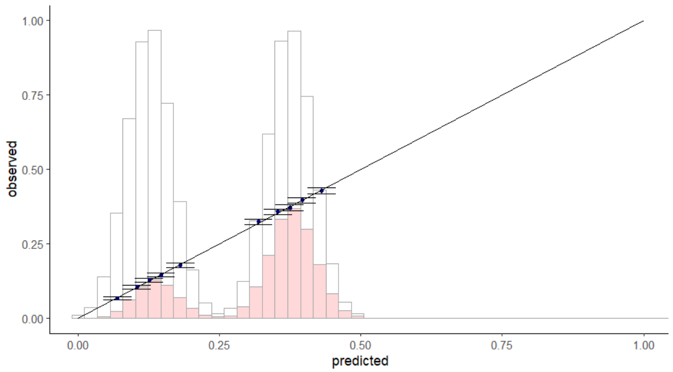

1.) Sensitivity analysis: score range 0.00-0.50

AUC	0.70 (0.69|0.70)
Brier-index	0.17 (0.17|0.17)
Brier-index scaled	0.09 (0.09|0.10)
R-Squared	0.09 (0.09|0.10)
NRI	0.67 (0.65|0.68)

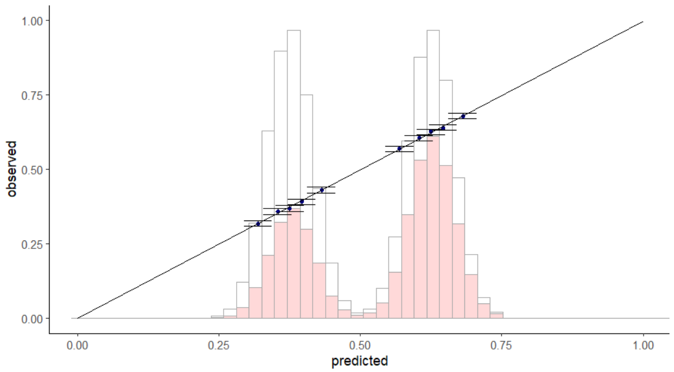

2.) Sensitivity analysis: score range 0.25-0.75

AUC	0.65 (0.64|0.65)
Brier-index	0.23 (0.23|0.23)
Brier-index scaled	0.07 (0.07|0.07)
R-Squared	0.07 (0.07|0.07)
NRI	0.50 (0.48|0.52)

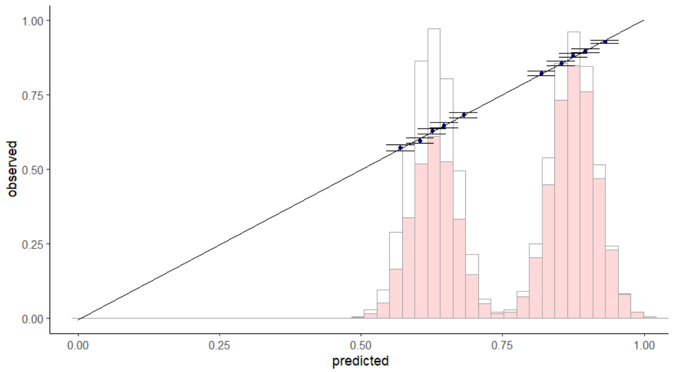

3.) Sensitivity analysis: score range 0.50-1.00

AUC	0.70 (0.69|0.70)
Brier-index	0.17 (0.17|0.17)
Brier-index scaled	0.09 (0.09|0.10)
R-Squared	0.09 (0.09|0.10)
NRI	0.67 (0.65|0.68)

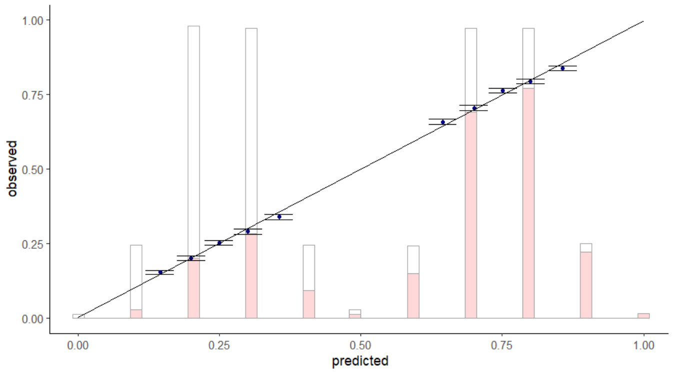

4.) Rounded score

AUC	0.79 (0.79|0.80)
Brier-index	0.18 (0.18|0.18)
Brier-index scaled	0.27 (0.26|0.28)
R-Squared	0.27 (0.27|0.28)
NRI	1.00 (0.99|1.02)

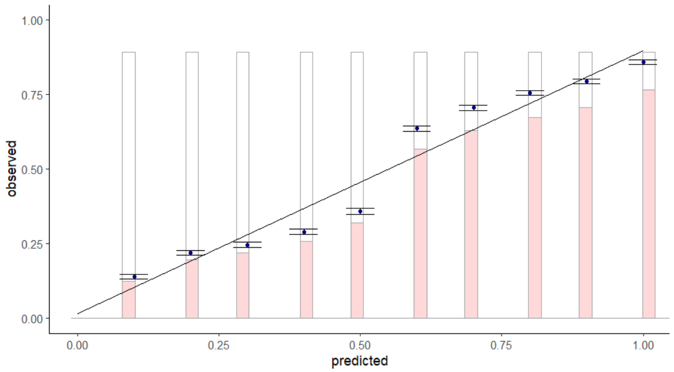

5.) Deciles

AUC	0.79 (0.79|0.80)
Brier-index	0.18 (0.18|0.19)
Brier-index scaled	0.26 (0.26|0.27)
R-Squared	0.26 (0.25|0.27)
NRI	0 (0|0)

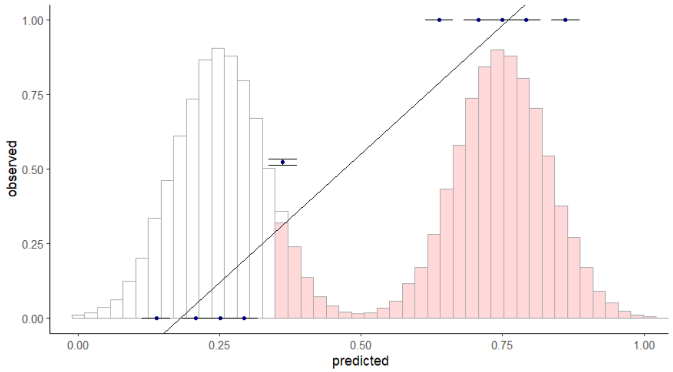

6.) Random threshold

AUC	1 (1|1)
Brier-index	0 (0|0)
Brier-index scaled	1.00 (1.00|1.00)
R-Squared	0.56 (0.00|0.91)
NRI	1.03 (0.00|2.00)

Supplementary Figure 3. Results for the sensitivity analyses for the bimodal normal distribution. The figures depict the histograms for the bimodal normal distribution across the sensitivity analyses with individuals with an event shaded in pink. Projected on top are the calibration plots between the observed and predicted incidence rates. Below each distribution are the performance results for each metric, with (minimum|maximum) range based on 1,000 bootstraps. For the ‘Score range’ analyses, the influence of the score range was assessed, by adapting the range of each distribution to span 0.00-0.50, 0.25-0.75, and 0.50-1.00 respectively. For the ‘Rounded score’ analyses, the influence of the score’s granularity was assessed, by rounding the risk scores to the nearest 0.1 after the events had been generated. For the “Deciles” analysis, individuals were categorized in 10 equal sized groups according to deciles of their risk score. For the “Random cut-off” analyses, for each iteration of the bootstrap, a random threshold was chosen above which all individuals experienced an outcome event. Abbreviations: AUC=Area Under the Curve, NRI=Net Reclassification Improvement

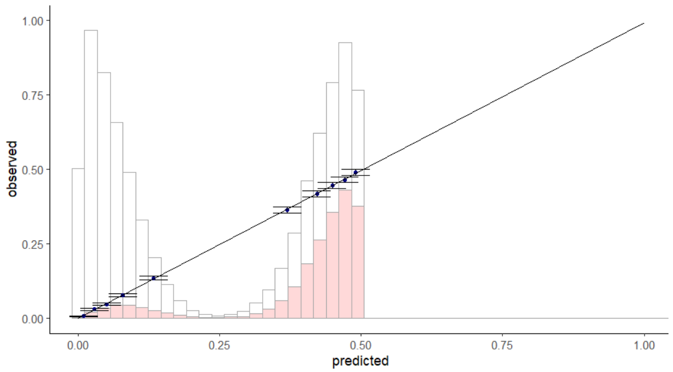

1.) Sensitivity analysis: score range 0.00-0.50

AUC	0.79 (0.78|0.79)
Brier-index	0.15 (0.15|0.15)
Brier-index scaled	0.20 (0.19|0.21)
R-Squared	0.20 (0.20|0.21)
NRI	1.01 (1.00|1.03)

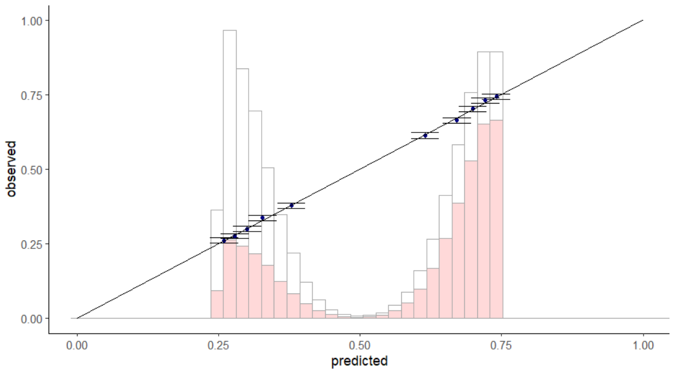

2.) Sensitivity analysis: score range 0.25-0.75

AUC	0.72 (0.71|0.72)
Brier-index	0.21 (0.21|0.21)
Brier-index scaled	0.15 (0.15|0.16)
R-Squared	0.15 (0.15|0.16)
NRI	0.76 (0.74|0.78)

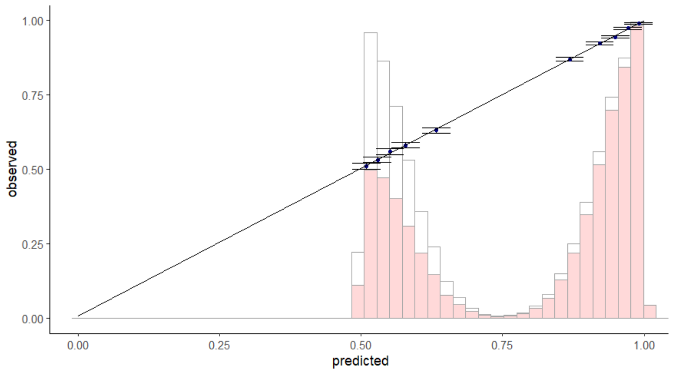

3.) Sensitivity analysis: score range 0.50-1.00

AUC	0.79 (0.78|0.79)
Brier-index	0.15 (0.15|0.15)
Brier-index scaled	0.20 (0.19|0.21)
R-Squared	0.20 (0.20|0.21)
NRI	1.01 (1.00|1.03)

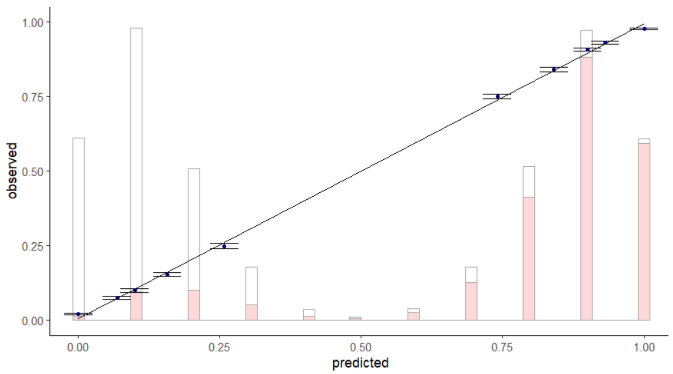

4.) Rounded score

AUC	0.93 (0.92|0.93)
Brier-index	0.10 (0.10|0.10)
Brier-index scaled	0.61 (0.60|0.61)
R-Squared	0.61 (0.60|0.62)
NRI	1.52 (1.51|1.53)

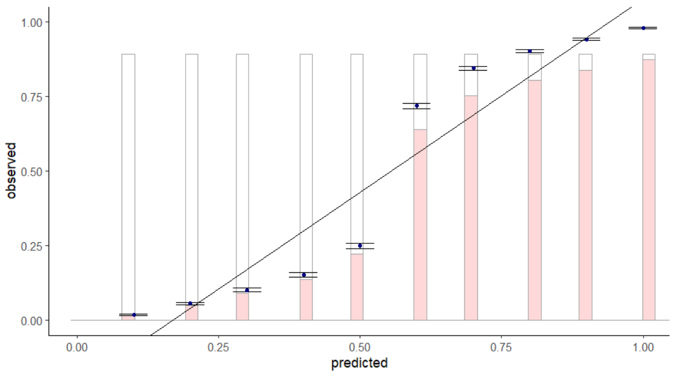

5.) Deciles

AUC	0.93 (0.93|0.92)
Brier-index	0.10 (0.10|0.10)
Brier-index scaled	0.59 (0.58|0.60)
R-Squared	0.55 (0.55|0.56)
NRI	0 (0|0)

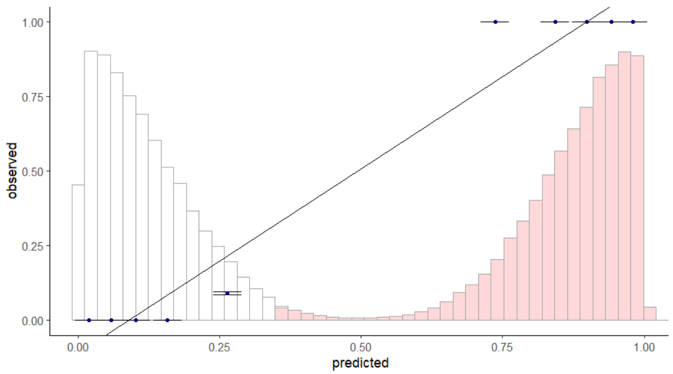

6.) Random threshold

AUC	1 (1|1)
Brier-index	0 (0|0)
Brier-index scaled	1.00 (1.00|1.00)
R-Squared	0.89 (0.09|0.95)
NRI	1.86 (0.31|2.00)

Supplementary Figure 4. Results for the sensitivity analyses for the bimodal extreme distribution. The figures depict the histograms for the bimodal extreme distribution across the sensitivity analyses with individuals with an event shaded in pink. Projected on top are the calibration plots between the observed and predicted incidence rates. Below each distribution are the performance results for each metric, with (minimum|maximum) range based on 1,000 bootstraps. For the 'Score range' analyses, the influence of the score range was assessed, by adapting the range of each distribution to span 0.00-0.50, 0.25-0.75, and 0.50-1.00 respectively. For the 'Rounded score' analyses, the influence of the score's granularity was assessed, by rounding the risk scores to the nearest 0.1 after the events had been generated. For the "Deciles" analysis, individuals were categorized in 10 equal sized groups according to deciles of their risk score. For the "Random cut-off" analyses, for each iteration of the bootstrap, a random threshold was chosen above which all individuals experienced an outcome event. Abbreviations: AUC=Area Under the Curve, NRI=Net Reclassification Improvement

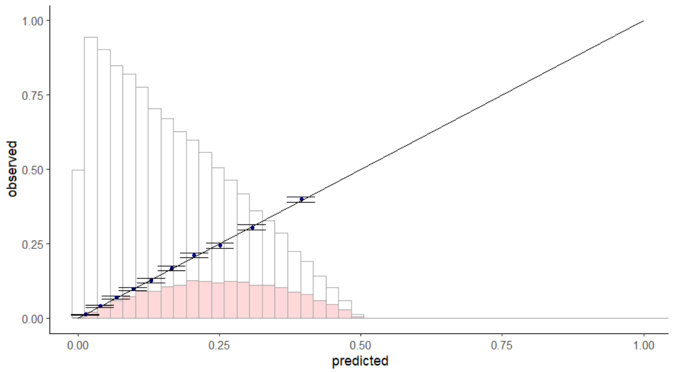

1.) Sensitivity analysis: score range 0.00-0.50

AUC	0.74 (0.74|0.74)
Brier-index	0.13 (0.12|0.13)
Brier-index scaled	0.09 (0.087|0.1)
R-Squared	0.10 (0.10|0.11)
NRI	0.71 (0.69|0.73)

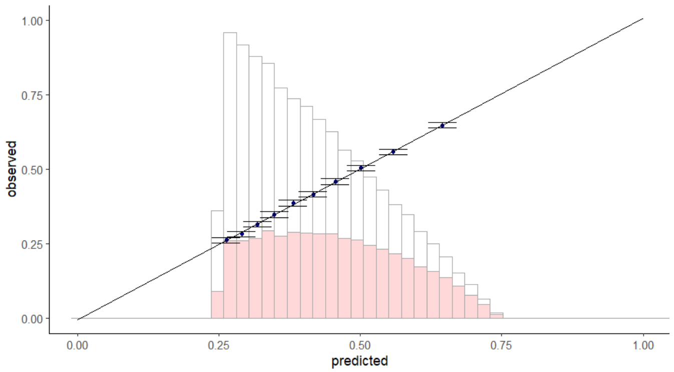

2.) Sensitivity analysis: score range 0.25-0.75

AUC	0.64 (0.63|0.64)
Brier-index	0.23 (0.23|0.23)
Brier-index scaled	0.06 (0.05|0.06)
R-Squared	0.06 (0.05|0.06)
NRI	0.41 (0.39|0.43)

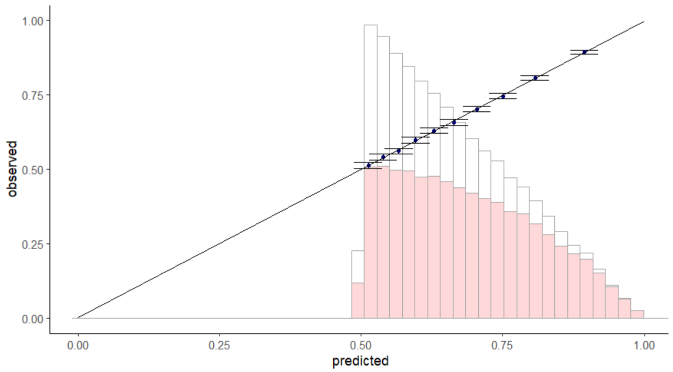

3.) Sensitivity analysis: score range 0.50-1.00

AUC	0.65 (0.64|0.66)
Brier-index	0.21 (0.21|0.21)
Brier-index scaled	0.06 (0.06|0.07)
R-Squared	0.06 (0.06|0.07)
NRI	0.45 (0.43|0.46)

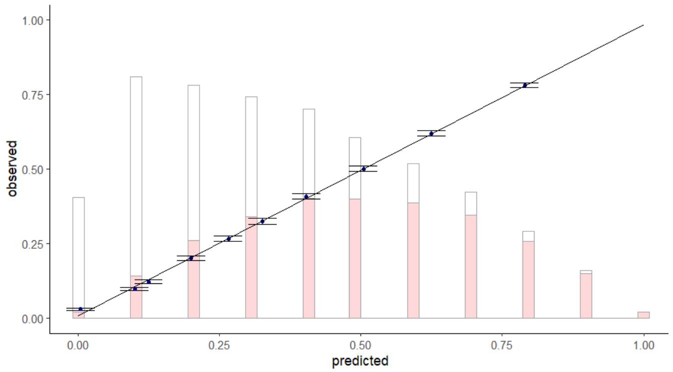

4.) Rounded score

AUC	0.80 (0.79|0.80)
Brier-index	0.17 (0.17|0.17)
Brier-index scaled	0.24 (0.24|0.25)
R-Squared	0.25 (0.24|0.25)
NRI	0.89 (0.87|0.90)

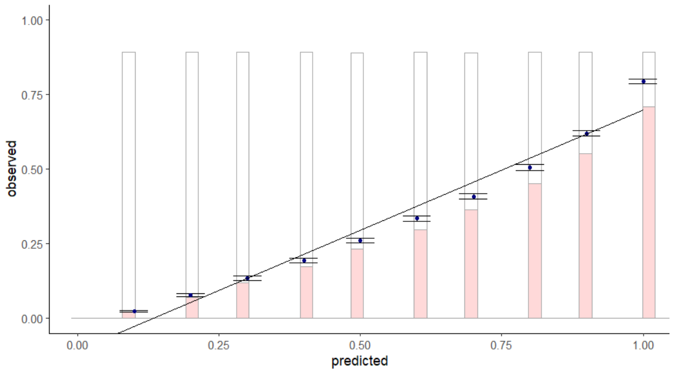

5.) Deciles

AUC	0.80 (0.79|0.80)
Brier-index	0.17 (0.17|0.17)
Brier-index scaled	0.24 (0.24|0.25)
R-Squared	0.24 (0.23|0.24)
NRI	0 (0|0)

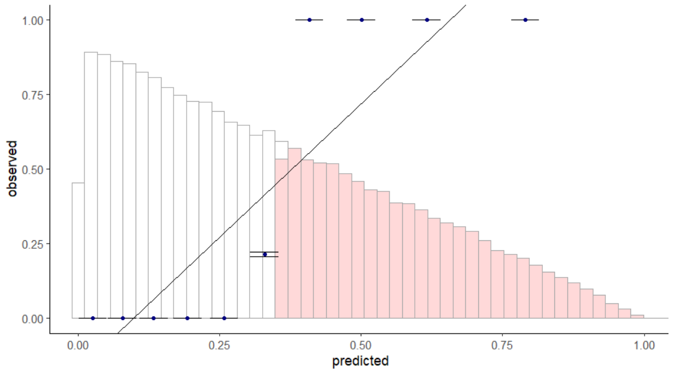

6.) Random threshold

AUC	1 (1|1)
Brier-index	0 (0|0)
Brier-index scaled	1.00 (0.98|1.00)
R-Squared	0.53 (0.00|0.72)
NRI	0.46 (0.00|1.94)

Supplementary Figure 5. Results for the sensitivity analyses for the uniform descending distribution. The figures depict the histograms for the uniform descending distribution across the sensitivity analyses with individuals with an event shaded in pink. Projected on top are the calibration plots between the observed and predicted incidence rates. Below each distribution are the performance results for each metric, with (minimum|maximum) range based on 1,000 bootstraps. For the 'Score range' analyses, the influence of the score range was assessed, by adapting the range of each distribution to span 0.00-0.50, 0.25-0.75, and 0.50-1.00 respectively. For the 'Rounded score' analyses, the influence of the score's granularity was assessed, by rounding the risk scores to the nearest 0.1 after the events had been generated. For the "Deciles" analysis, individuals were categorized in 10 equal sized groups according to deciles of their risk score. For the "Random cut-off" analyses, for each iteration of the bootstrap, a random threshold was chosen above which all individuals experienced an outcome event. Abbreviations: AUC=Area Under the Curve, NRI=Net Reclassification Improvement

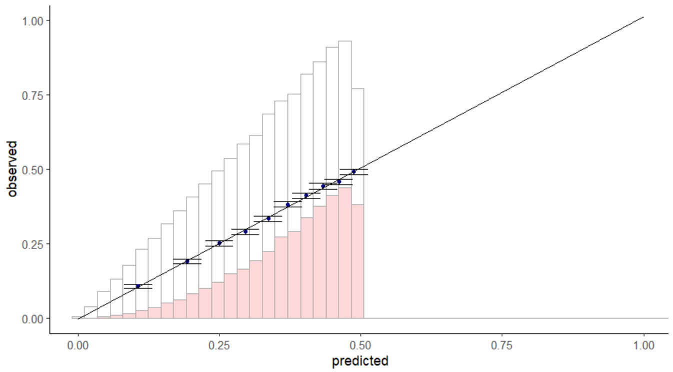

1.) Sensitivity analysis: score range 0.00-0.50

AUC	0.65 (0.65|0.65)
Brier-index	0.21 (0.21|0.21)
Brier-index scaled	0.06 (0.06|0.07)
R-Squared	0.06 (0.06|0.07)
NRI	0.45 (0.43|0.73)

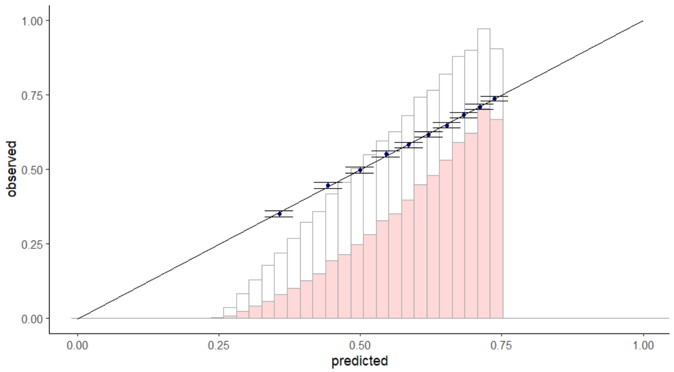

2.) Sensitivity analysis: score range 0.25-0.75

AUC	0.64 (0.63|0.64)
Brier-index	0.23 (0.23|0.23)
Brier-index scaled	0.06 (0.05|0.06)
R-Squared	0.06 (0.05|0.06)
NRI	0.41 (0.39|0.43)

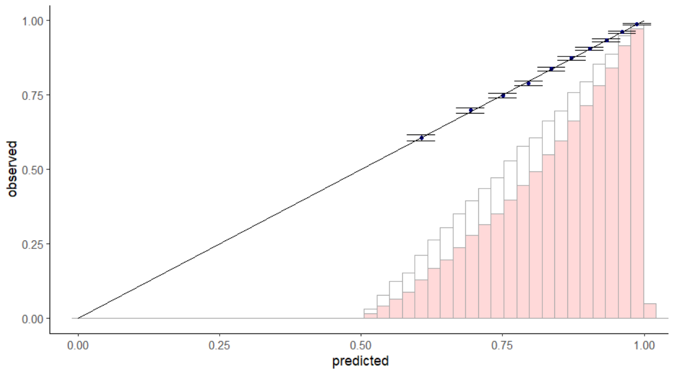

3.) Sensitivity analysis: score range 0.50-1.00

AUC	0.74 (0.74|0.74)
Brier-index	0.13 (0.12|0.13)
Brier-index scaled	0.09 (0.09|0.10)
R-Squared	0.10 (0.10|0.10)
NRI	0.71 (0.69|0.46)

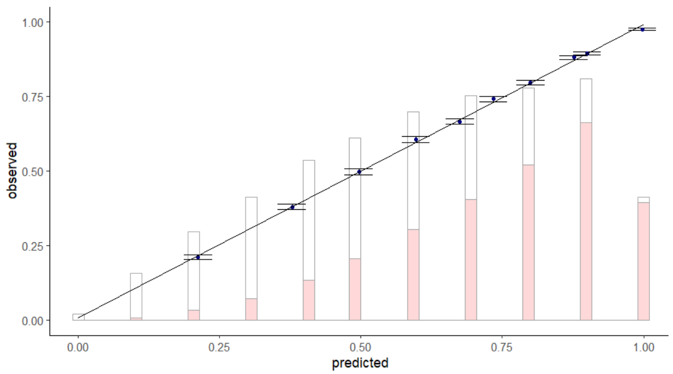

4.) Rounded score

AUC	0.80 (0.79|0.80)
Brier-index	0.17 (0.17|0.17)
Brier-index scaled	0.24 (0.24|0.25)
R-Squared	0.25 (0.24|0.25)
NRI	0.89 (0.87|0.90)

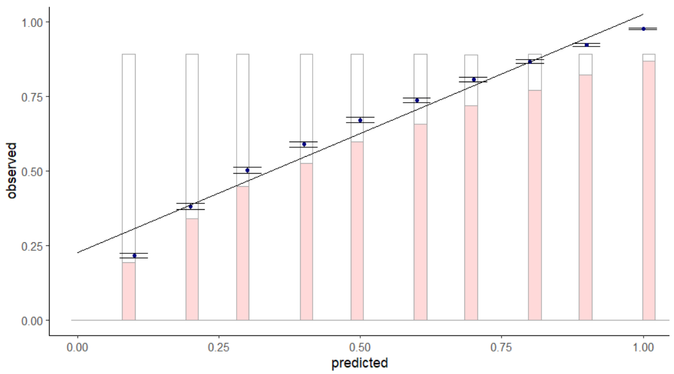

5.) Deciles

AUC	0.80 (0.79|0.80)
Brier-index	0.17 (0.17|0.17)
Brier-index scaled	0.24 (0.23|0.25)
R-Squared	0.24 (0.23|0.24)
NRI	0 (0|0)

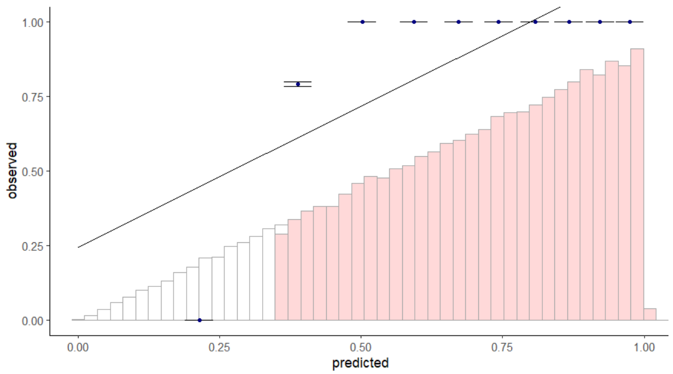

6.) Random threshold

AUC	1 (1|1)
Brier-index	0 (0|0)
Brier-index scaled	1.00 (0.99|1.00)
R-Squared	0.52 (0.01|0.72)
NRI	0.46 (0.00|1.94)

Supplementary Figure 6. Results for the sensitivity analyses for the uniform ascending distribution. The figures depict the histograms for the uniform ascending distribution across the sensitivity analyses with individuals with an event shaded in pink. Projected on top are the calibration plots between the observed and predicted incidence rates. Below each distribution are the performance results for each metric, with (minimum|maximum) range based on 1,000 bootstraps. For the ‘Score range’ analyses, the influence of the score range was assessed, by adapting the range of each distribution to span 0.00-0.50, 0.25-0.75, and 0.50-1.00 respectively. For the ‘Rounded score’ analyses, the influence of the score’s granularity was assessed, by rounding the risk scores to the nearest 0.1 after the events had been generated. For the “Deciles” analysis, individuals were categorized in 10 equal sized groups according to deciles of their risk score. For the “Random cut-off” analyses, for each iteration of the bootstrap, a random threshold was chosen above which all individuals experienced an outcome event. Abbreviations: AUC=Area Under the Curve, NRI=Net Reclassification Improvement

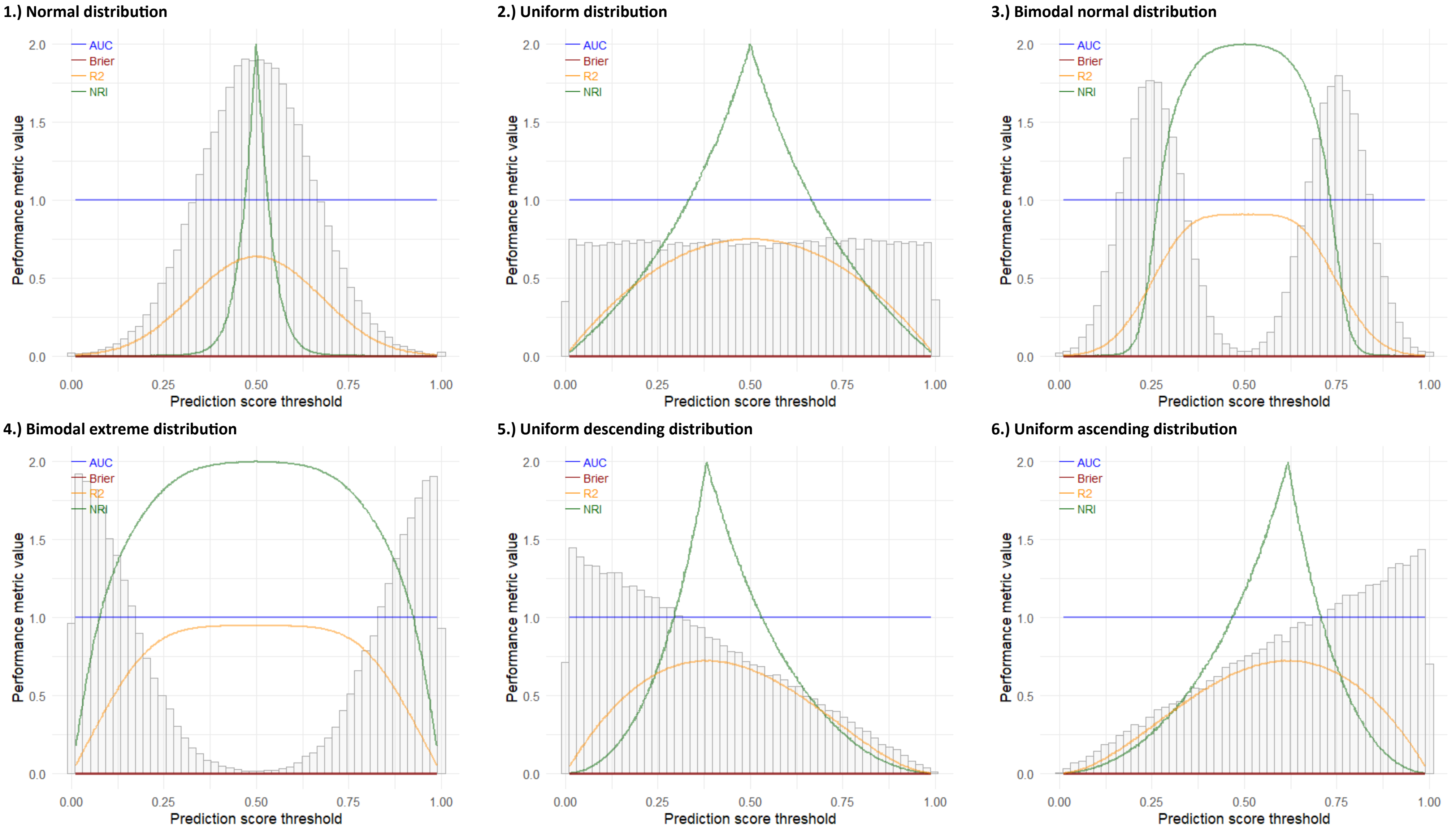

Supplementary Figure 7. Performance metric values for the different distributions according to the threshold above which everyone experiences an event. Figures display the histogram according to which the risk score is distributed. The lines represent the value for each performance metric (y-axis) based on the threshold above which everyone experiences an event (x-axis). For the AUC and Brier, scores are always the optimum values (1 and 0 respectively), regardless of the threshold and distribution. For the NRI and R-squared, the values differ with different thresholds, with the optimum value at the 50th percentile. For the NRI, the values vary across the whole range, regardless of distribution. For the R-squared, values vary up to a maximum, which depends on the distribution. The Brier scaled score is not displayed as it practically equals the AUC score in this scenario being consistently 1.

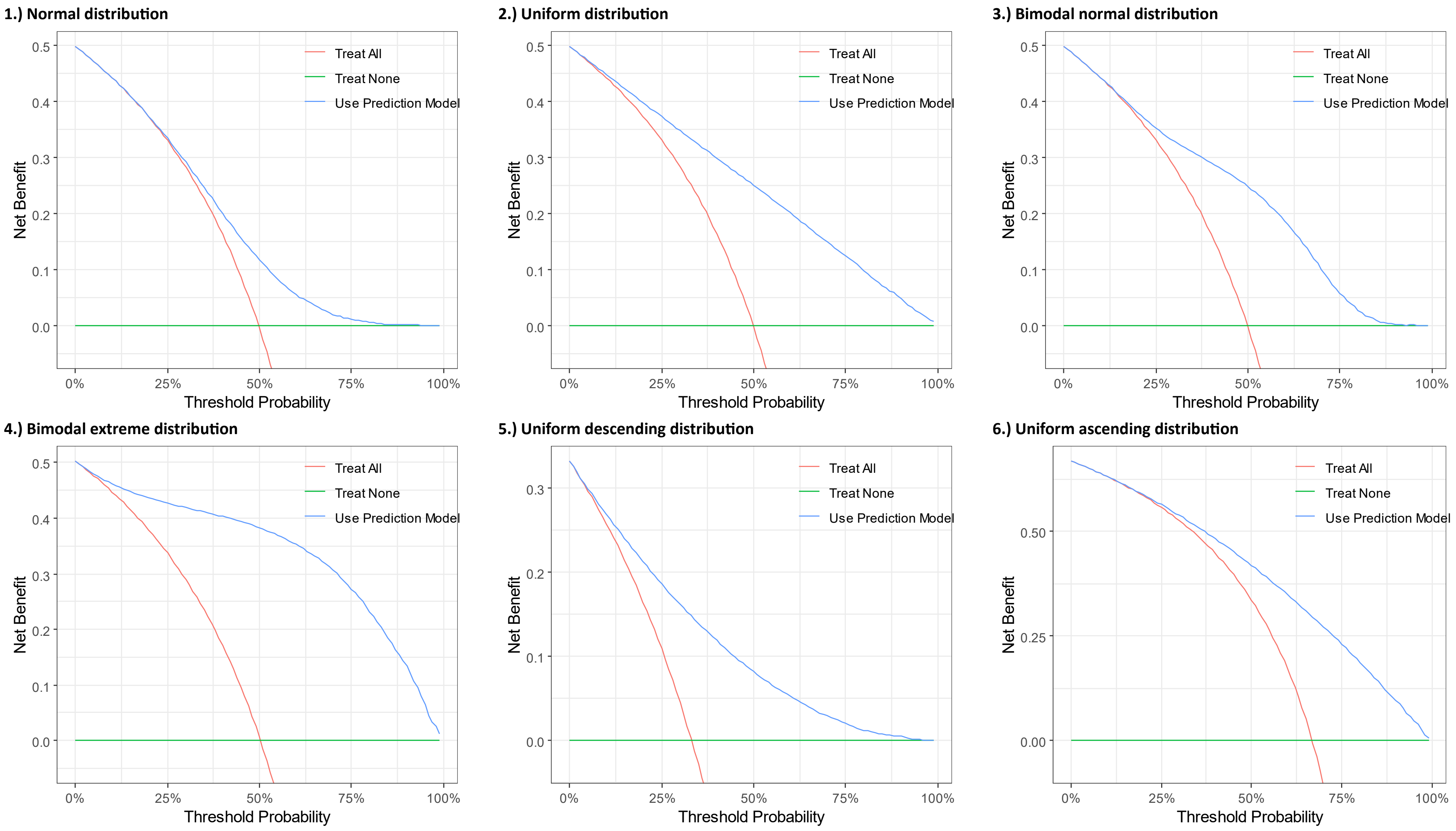

Supplementary Figure 8. Decision Curve Analysis (DCA) graphs for the different distributions. The DCA graphs can be used to evaluate whether using a clinical model or test to make decisions is better than just treating anyone or treating no one. The y-axis depicts the “Net Benefit”, which is the difference between the proportion of true positives and weighted proportion of false positives (“weighted” because the higher the threshold, the less likely that false positives are acceptable, else the threshold would be set lower). The x-axis depicts the threshold of the predicted risk at which patients receive treatment. The lines depict the benefit of treating all participants (red), the benefit of treating no one (green), and the the benefit of treating everyone with a risk exceeding the threshold on the x-axis. A model is generally considered clinically useful if its net benefit is higher than both 'treat all' and 'treat none' across a range of plausible risk thresholds. The clinical value of the model is greatest at the threshold where the gap between the model's net benefit and the next best strategy is widest.

Authors (reference #)	Journal	Year	Scores	Goal	AUC	Focus on AUC	P/O%	Calibration Slope	Calibration qualitative	Calibration mentioned
Wernly (64)	European journal of preventive cardiology	2025	SCORE2	Validate/compare new score	Yes	-	-	-	-	-
Belahnech (63)	European journal of preventive cardiology	2025	SCORE2	Validation	Yes	-	Yes	-	Yes	Yes
Enguita-Germán (62)	European journal of preventive cardiology	2025	SCORE2	Validation	Yes	-	-	-	-	Yes
Xie (61)	European Journal of Epidemiology	2025	SCORE2	Validate/compare new score	Yes	Yes	-	-	-	-
Gynnild (60)	European journal of preventive cardiology	2025	SCORE2	Validate/compare new score	Yes	-	Yes	-	Yes	Yes
de la Harpe (**59**)	European journal of preventive cardiology	2025	SCORE2	Validate/compare new score	Yes	-	Yes	-	Yes	Yes
Alfaraj (58)	European journal of preventive cardiology	2025	SCORE2	Validation	Yes	-	Yes	Yes	Yes	Yes
Lopez-Lopez (57)	European journal of preventive cardiology	2025	SCORE2	Validation	Yes	Yes	-	-	Yes	Yes
Xie (56)	European journal of preventive cardiology	2025	SCORE2	Validate/compare new score	Yes	Yes	-	-	-	-
Fu (55)	European journal of preventive cardiology	2025	SCORE2	Validation	Yes	-	Yes	-	Yes	Yes
Hughes (54)	Clinical Rheumatology	2025	QRISK3	Validation	Yes	Yes	-	-	-	-
SCORE2 Asia-Pacific group (53)	European Heart Journal	2025	SCORE2	Validate/compare new score	Yes	Yes	-	-	-	-
Yacaman Mendez (52)	European journal of preventive cardiology	2025	SCORE2	Validate/compare new score	Yes	-	-	-	-	-
Xie (51)	Cardiovascular Diabetology	2025	SCORE2	Validate/compare new score	Yes	Yes	-	-	-	-
van Apeldoorn (50)	International Journal of Cardiology	2024	SCORE2	Validate/compare new score	Yes	Yes	-	-	-	Yes
Temtem (49)	Rev Port Cardiol.	2024	SCORE2	Validation	Yes	-	-	-	-	Yes
Amegadzie (48)	Thorax	2024	QRISK3	Validation	-	-	Yes	-	-	Yes
Svinin (47)	PLoS One	2024	SCORE2	Validation	-	-	-	-	-	-
Hippisley-Cox (24)	Nature medicine	2024	QRISK3, SCORE2	Validate/compare new score	Yes	Yes	-	-	-	-
Sud (25)	European journal of preventive cardiology	2024	SCORE2	Validation	Yes	-	Yes	-	Yes	Yes
van Trier (26)	European journal of preventive cardiology	2024	SCORE2	Validation	Yes	-	Yes	-	Yes	Yes
Jalepalli (27)	Family medicine and community health	2024	QRISK3	Validate/compare new score	Yes	Yes	-	-	-	-
Temtem (28)	Revista portuguesa de cardiologia	2024	SCORE2	Validation	Yes	-	-	-	Yes	Yes
Hughes (29)	Rheumatology	2023	QRISK3	Validation	Yes	Yes	-	-	Yes	Yes
Kasim (30)	The Lancet regional health. Western Pacific	2023	SCORE2	Validation	Yes	-	Yes	-	Yes	Yes
Parsons (13)	Heart	2023	QRISK3	Validation	Yes	Yes	Yes	-	Yes	Yes
Huang (42)	European journal of preventive cardiology	2023	QRISK3	Validate/compare new score	Yes	Yes	-	-	-	-
Livingstone (31)	BMC cardiovascular disorders	2023	QRISK3	Validation	Yes	-	-	-	Yes	Yes
Jordan (32)	European journal of preventive cardiology	2023	QRISK3	Validate/compare new score	Yes	-	-	Yes	Yes	Yes
Kist (33)	EClinicalMedicine	2023	SCORE2	Validation	Yes	-	Yes	-	Yes	Yes
Ahmed (34)	Preventive medicine reports	2023	QRISK3	Validation	Yes	-	-	-	Yes	Yes
Tseng (43)	BMC medicine	2023	QRISK3	Validate/compare new score	Yes	Yes	-	-	-	-
Matsushita (44)	European journal of preventive cardiology	2023	SCORE2	Validate/compare new score	Yes	Yes	-	-	-	-
Amegadzie (35)	Thorax	2023	QRISK3	Validate	-	-	Yes	-	Yes	Yes
Wamil (45)	Scientific reports	2023	QRISK	Validate/compare new score	Yes	Yes	-	-	-	-

Hageman (46)	European journal of preventive cardiology	2023	SCORE2	Validate/compare new score	Yes	Yes	-	-	-	Yes
SCORE2-DM workgroup (39)	European heart journal	2023	SCORE2	Validate/compare new score	Yes	Yes	-	-	Yes	Yes
Pezel (36)	JACC. Cardiovascular imaging	2022	QRISK3	Validate/compare new score	Yes	Yes	-	-	-	-
Livingstone (37)	BMC medicine	2022	QRISK3	Validate/compare new score	Yes	-	-	-	Yes	Yes
Steinfeldt (38)	The Lancet. Digital health	2022	QRISK3	Validate/compare new score	Yes	-	-	-	Yes	Yes
SCORE2 workgroup (2)	European heart journal	2021	SCORE2	Validate/compare new score	Yes	-	-	-	-	Yes
Livingstone (12)	The Lancet. Healthy longevity	2021	QRISK3	Validated	Yes	-	-	-	Yes	Yes
SCORE2-OP workgroup (40)	European heart journal	2021	SCORE2	Validation	Yes	-	-	-	Yes	Yes
Perry (41)	Acta psychiatrica Scandinavica	2020	QRISK3	Validation	Yes	-	-	-	Yes	Yes
Hippisley-Cox (1)	BMJ	2017	QRISK3	Validate/compare new score	Yes	-	-	-	Yes	Yes

Supplementary Table 1. Mentions of AUC and calibration related performance in abstracts of validation studies for the QRISK3 and SCORE2 cardiovascular risk scores. We searched pubmed for "(QRISK3 OR SCORE2) AND validat*" on August 26th 2025 and retrieved 119 results. Results only represent the information in the abstracts. **AUC** denotes whether results reported numbers for the AUC or C-statistic; **Focus on AUC** denotes whether the abstract predominantly described the AUC; P/O% denotes whether the abstract reported percentages for predicted versus observed incidence rates; **Calibration slope** denotes whether the abstract reported a number for the calibration slope; **Calibration qualitative** denotes whether the abstract commented on the calibration qualitatively e.g. 'good calibration'; **Calibration mentioned** denotes whether performance relating to calibration was at all mentioned in the abstract. **Reference #** refer to reference numbers in the main manuscript.
